# Embryoid research calls for reassessment of legal regulations

**DOI:** 10.1186/s13287-021-02442-2

**Published:** 2021-06-19

**Authors:** Markus Hengstschläger, Margit Rosner

**Affiliations:** Institute of Medical Genetics, Center for Pathobiochemistry and Genetics, Medical University of Vienna, Währinger Straße 10, 1090 Vienna, Austria

**Keywords:** Embryo, Embryoid, Ethics, Human embryonic stem cells, Law

## Abstract

It is known that in countries, in which basic research on human embryos is in fact prohibited by law, working with imported human embryonic stem cells (hESCs) can still be permitted. As long as hESCs are not capable of development into a complete human being, it might be the case that they do not fulfill all criteria of the local definition of an embryo. Recent research demonstrates that hESCs can be developed into entities, called embryoids, which increasingly could come closer to actual human embryos in future. By discussing the Austrian situation, we want to highlight that current embryoid research could affect the prevailing opinion on the legal status of work with hESCs and therefore calls for reassessment of the regulations in all countries with comparable definitions of the embryo.

## Background

In each country of the world, laws regulating human embryo research are rooted in national politics, religion, and history. Accordingly, one can assume that the Austrian Reproductive Medicine Act was made under the influence of the sorrowful but still so important memory of the unethical human medical research conducted during the Second World War. In Austria, basic research on human embryos is prohibited. In contrast, many other countries worldwide permit human embryo research, e.g., on surplus in vitro fertilization embryos, until day 14 post fertilization.

The Austrian law does not contain a precise definition of an embryo, but states that “fertilized egg cells and the cells developed from them,” when they are “viable cells” in the sense of “cells capable of development,” may not be used for purposes other than medically assisted reproduction [1]. According to the prevailing juridical opinion, only cells which meet the first definition and additionally are capable of development into a complete human being (a complete person) are covered by this prohibition. Consequently, research using pluripotent human embryonic stem cells (hESCs), which have been isolated outside the territorial scope of the Austrian legislation and are considered to harbor the potential to only develop into specific tissues, is permissible [2]. In Austria different research groups, including ours [3], are working with imported hESC lines.

## Current developments in human embryoid research

Over the last years, it has been demonstrated that hESCs can be driven to self-organized development of so called embryoids with embryo-like features. Meanwhile, several different stem cell-based models of embryos recapitulating different aspects of early human development exist. Recently, it was highlighted that one general term should universally be used for these models, when engaging with the public and the media, to avoid confusion and controversy. In this context the suffix “oid” is well known to refer to similarity without identity. Additionally, a more detailed nomenclature should be established to specifically describe the utilities and limitations of the different models for scientists and policymakers [4].

Very recently, two groups reported the stem cell-based in vitro establishment of human blastocyst-like structures, containing all developmental lineages. Although the authors acknowledge that these blastoid models still exhibit relevant limitations, the development of integrated human pre-implantation/early post-implantation embryo models containing all founding cell lineages of the fetus and its supporting tissues will have enormous impact for a more comprehensive understanding of the early steps of human embryogenesis [5, 6].

Post-implantation human development can be investigated by already earlier established embryoids, which, however, lack lineages associated with the trophectoderm, hypoblast, or both. These models include gastrulation micropatterned colonies, the post-implantation amniotic sac embryoid, and the asymmetric early post-implantation epiblasts [7]. And recently, the first three-dimensional model mimicking human embryogenesis beyond the 14 day boundary has been generated [8].

All approaches to investigate the early steps of embryogenesis used so far exhibit specific limitations. Human embryos donated from in vitro fertilization patients often harbor aneuploidies and genetic modification approaches are hindered by ethical and technical barriers. Ethical concerns, legal constraints, and the limited conservation of molecular processes confine studies using non-human primate embryos. And finally, due to significant interspecies differences, knowledge derived from investigations on mouse embryos can also not necessarily directly be assigned to humans. Human embryoids can be produced in large quantities and can be exposed to diverse experimental manipulations enabling statistical analyses. Gene editing approaches can be used to develop innovative embryo models for the molecular investigation of normal and pathological human development. There is good reason to hope that the rapidly growing field of embryoid research will allow to draw a more comprehensive picture of human embryogenesis, what is of highest relevance for infertility treatments, disease modeling, and drug discovery [9].

## Ethical and legal considerations

To date, the existing embryo models cannot develop into a human being. However, it is already under debate how close such models, including all cell types of an early human conceptus, can come to the natural human embryo in future [10]. Notably, the ban on the usage of stem-cell-based entities for reproductive purposes, particularly the implantation of embryoids into human uteri, has already been demanded [11]. Since the upcoming generations of embryoids will increasingly come closer to actual human embryos, it is crucial to anticipate the ethical discussion about their moral status and to develop appropriate guidelines and national laws for the related research reflecting justified ethical concerns. In this context, it is of utmost importance to reassess the currently used embryo definitions, which form the basis for the different national legal regulations. Whenever ethical reflections and legal restrictions aim to refer to the concept of the potentiality to develop into a human being, one will need to consider the according degrees of the developmental potential of embryoids. And furthermore, the discussion whether the 14-day rule can meaningfully be applied to embryoids must be initiated [12, 13].

Next to all these considerations already under discussion, we would like to highlight another relevant aspect in this context. Given that in future it becomes possible to develop human embryoids, which could be interpreted to qualify as human embryos, this could have broad implications for hESC research in general. As an ultimate consequence of such a development, in Austria, hESCs could be interpreted as “developed from fertilized egg cells,” which are then also “cells capable of development.” Obviously, any research attempt using hESCs, which in this scenario could be argued to meet both definitions, could consequently be interpreted to be prohibited in Austria. Many countries do not include any definitions of an embryo in their guidelines or laws. And the spectrum of national definitions exhibits substantial country-specific variation. Besides Austria in, e.g., Belgium, Netherlands, or Germany, the capability of the development into a human being is at least part of the criteria included into the definition of an embryo [14]. In Belgium, the embryo is defined as “the cell or the organic set of cells capable, as they develop, of becoming a human being.” In Netherlands, the embryo is defined as a “cell or a set of cells with the capacity to grow into a human.” And also, e.g., in Germany, the potentiality to develop into a human being is, beside other aspects, part of the definition: “Embryo is any totipotent cell which, if the necessary conditions are met, is able to divide and develop into an individual” ([14] and references therein). Not only in these countries, but in general, ethicists, policymakers, and regulators should review to which extent the actual progress regarding embryoid research could have implications for the legal evaluation of embryonic stem cell research.

## Conclusions

There is no doubt that human embryoids constitute a very promising tool paving the way to a more comprehensive understanding of human embryogenesis and pathologies. And it is also important to acknowledge that the current scientific progress regarding stem cell-based human embryo models invokes a variety of ethical questions with putative consequences for a realignment of legal regulations. We here want to draw attention to the fact that in light of the current developments, reassessments of specific legal regulations might become indispensable to avoid negative regulatory effects on human embryonic stem cell research.

## Data Availability

Not applicable.

## Abbreviation

hESCs: Human embryonic stem cells
